# Long term effectiveness of inactivated vaccine BBIBP-CorV (Vero Cells) against COVID-19 associated severe and critical hospitalization in Morocco

**DOI:** 10.1371/journal.pone.0278546

**Published:** 2022-12-07

**Authors:** Jihane Belayachi, Majdouline Obtel, Abdelkader Mhayi, Rachid Razine, Redouane Abouqal

**Affiliations:** 1 Acute Medical Unit, Ibn Sina University Hospital, Rabat, Morocco; 2 Laboratory of Biostatistics, Clinical, and Epidemiological Research, Faculty of Medicine and Pharmacy, Department of Public Health, Mohamed V University in Rabat, Rabat, Morocco; 3 Laboratory of Community Health (Public Health, Preventive Medicine and Hygiene), Faculty of Medicine and Pharmacy, Department of Public Health, Mohamed V University in Rabat, Rabat, Morocco; 4 Department of Informatics, Ministry of Health and Social Protection, Rabat, Morocco; The University of Hong Kong, CHINA

## Abstract

**Background:**

We provide national estimates of the real-world Vaccine effectiveness (VE) based on nationally available surveillance data. The study aimed to estimate the effectiveness of the inactivated Covid-19 vaccine BBIBP-CorV (Vero Cells) Sinopharm vaccine currently deployed in Morocco against SARS- CoV-2 severe disease/ hospitalization” within 9 months after vaccination.

**Methods:**

We conducted a test-negative, case-control study among a population aged 18 years or older who were tested by rt-PCR for SARS-CoV-2 infection from February to October 2021 in Morocco. From the national laboratory COVID-19 database; we identified cases who were rt-PCR positive amongst severe and critical COVID-19 cases and controls who had a negative rt-PCR test for SARS-CoV-2. From the national vaccination register (NVR); individuals vaccinated with COVID-19 Vaccine (Vero Cell) and those unvaccinated were identified and included in the study. The linkage between databases was conducted for the study of Vaccination status based on the timing of the vaccine receipt relative to the SARS-CoV-2 rt-PCR test date. For each person, who tested positive for SARS-CoV-2, we identified a propensity score-matched control participant who was tested negative. We estimated vaccine effectiveness against SARS- CoV-2 severe disease/ hospitalization using conditional logistic regression.

**Results:**

Among 12884 persons who tested positive and 12885 propensity score-matched control participants, the median age was 62 years, 47.2% of whom were female. As a function of time after vaccination of second dose vaccination, vaccine effectiveness during the first month was 88% (95% CI, 84–91), 87% (95% CI: 83–90) during the second and third month, 75% (95% CI: 67–80) during the fourth month, 61% (95% CI: 54–67) during the fifth month, and 64% (95% CI: 59–69) beyond the sixth month. VE remained high and stable during the first three months in the two-age subgroup. In the fourth month, the VE in the older population aged 60 years and above (64%) was reduced by 20 points compared to VE in the younger population (84%).

**Conclusion:**

A Sinopharm vaccine is highly protective against serious SARS-CoV-2 infection under real-world conditions. Protection remained high and stable during the first three months following the second dose and decreases slightly beyond the fourth month especially beyond 60 years.

## Introduction

As of January 24, 2022, the COVID-19 pandemic has resulted in more than 330 million cases and more than 5.5 million deaths worldwide, including 1.06 million cases and 15 025 deaths in the Kingdom of Morocco [1].

The development and deployment of sustainably effective vaccines amongst susceptible and at-risk populations remain key to end the pandemic. Since January 2021, four COVID-19 vaccines—BNT162b2 (Pfizer-BioNTech), Ad26.COV2.S (Johnson & Johnson-Janssen), the ChAdOx1 nCoV-19 (AZD1222; Oxford-AstraZeneca) and BBIBP-CorV (Vero Cells) Sinopharm; received emergency use authorization in Morocco based on short-term safety and efficacy against COVID-19 and were deployed in Morocco shortly thereafter.

In January 2021, a nationwide vaccination campaign was launched in Morocco; to administer COVID-19 vaccines for people aged 18 years and above. Guiding principles of Moroccan Strategy for Vaccination against sars-cov-2 was equity, solidarity, free, volunteering and transparency. Proximity deployment with mass and progressive vaccination; effective scientific transparent communication with a Smart Vaccination Campaign. Morocco ordered a total of 66 million vaccine doses to cover 33 million beneficiaries representing more than 80% of the population. The vaccination campaign was officially launched on January 28, 2021. An extensive operational system is being mobilized comprising 2,880 designated primary healthcare establishments as well as a large number of associated vaccine stations. A total of 25,631 individuals have been mobilized on the ground, mainly through 3,047 fixed stations and more than 10,000 mobile points in the 12 regions of the kingdom [1]. To prepare for this complex and large-scale vaccination campaign and to implement follow-up activities, the Ministry of health and social protection has setup a multiple-component digital system comprising a vaccination registry, stock, and logistics management facilities, and a portal for tracking side effects. A platform named “liqah” (“vaccine” in Arabic) has concurrently been set up (www.liqahcorona.ma), which allows doctors to have direct contact with citizens.

As of January 2022, one year after the start of the vaccination campaign it is estimated that nearly 70% of the target population (30,000,000) has received at least one dose of vaccine, with Sinopharm amongst 72% of vaccine receipt [2].

Morocco recorded its first case of COVID-19 on March 2, 2020. Subsequently, the authorities declared a state of health emergency on March 20 even though the number of daily cases in the country was only about 10 at this time. Since then, the pandemic’s evolution in Morocco has evidenced a controlled trend, with an average daily growth rate of around 5.5%, a prevalence of <1%, and an average fatality rate of 4% during the lockdown period from March to May 2020. At the end of 3 months of strict confinement, the epidemiological indicators favored a progressive zone-wise process of deconfinement that commenced on June 10, 2020. However, immediately after this process began, the pandemic escalated rapidly, with multiple industrial and family clusters emerging, which increased the incidence and prevalence rate. The Kingdom of Morocco is divided into 12 administrative regions. During this epidemic, in each region patients infected with SARS-CoV-2 are treated in dedicated health facilities through a three level of health-care system. Primary Health Care Establishments ensure; a) The Identification of suspected cases and their referral to local reference structure, b) Identification of suspected cases and their referral to local reference structures, c) Monitoring of cases treated at home, d) Participation with the Rapid Intervention Teams in the investigation of clusters and confirmed cases in order to identify contacts and direct them to reference structures according to their state of health. COVID-19 local reference structures ensure; a) Identification and triage of cases referred, the private sector and other stuctures; b) Referral of cases with risk factors and/or showing signs of seriousness to hospital care structures; c) Samples for PCR or carrying out the Antigen Test on site if indicated; d) Prescription and dispensing of drugs for cases eligible for home treatment. Secondary and tertiary Hospital structures admitted patients eligible for possible hospitalization according to the circuit established for this purpose. Hospital Admission criteria included: a) SpO2 < 92% without signs of severe respiratory failure; b) Positive COVID patient with a clinical condition requiring continuous monitoring (decompensated comorbidity, etc.) c) Need for invasive or non-invasive ventilation (SpO2 < 90% with signs of severe respiratory failure, SpO2 < 92% under 8 l/min of O2 or under a high concentration mask for more than 1 hour, etc.); d) Presence of other acute failures involving the vital prognosis.

Phase III trials reported high vaccine efficacy against SARS-CoV-2 infection with, two doses of the inactivated COVID-19 vaccine showing 72.8% and 78% efficacy against symptomatic coronavirus disease 2019 and 79% (26–94) efficacy against severe disease or hospitalization 21 days after the first dose in people aged 18 years and above. However, Phase III trials data provided sparse information on the impact of vaccination on transmission of SARS-CoV-2 infection and did not take into account different population groups; and data shown are on protection against SARS-CoV-2 ancestral strain.

Since the start of the outbreak, Morocco has experienced three epidemic waves corresponding to three periods of appearance of SARS-CoV2 variants of concerns from 02/03/20 to 01/05/22. The fisrt wave (from February to May 2021) noted the circulation of the Alpha variant (B.1.1.7).” the second wave (from July to September 2021) noted the dominance of circulation of Delta (B.1.617.2) variant (80%). The third wave is characterized, during early January 2022, by a circulation of Omicron (B.1.1.529) variant predominantly (70%).

Data on the real-world effectiveness of inactivated COVID-19 vaccine BBIBP-CorV (Vero cells) are limited. The effectiveness and duration of protection offered by the inactivated COVID-19 vaccine BBIBP-CorV (Vero cells) against serious or critical hospitalizations associated with COVID-19 are not known in Morocco.

We provide national estimates of the real-world effectiveness based on surveillance data available in the Kingdom of Morocco, which has a population of approximately 37 million inhabitants. This study aimed to estimate the effectiveness of the inactivated COVID-19 vaccine BBIBP-CorV (Vero Cells) Sinopharm currently deployed in the Kingdom of Morocco to reduce the risks of severe hospitalization associated with SARS-CoV-2 infection 9 months after completing the vaccination.

## Methods

### Study design

We estimate the real-world vaccine effectiveness (VE) over time after administration of two doses of inactivated COVID-19 vaccine BBIBP-CorV (Vero Cells) Sinopharm against COVID-19-associated severe or critical hospitalized cases using a test-negative, case-control study design comparing the odds of a positive SARS-CoV-2 test result between vaccinated and unvaccinated patients.

Eligible medical encounters were defined as those among adults aged 18 years and above who had a reverse transcription real-time polymerase chain reaction (rt-PCR) test for SARS-CoV-2 infection between February 1 2021 and October 1 2021. The start of the study period corresponded to 14 days after the first individuals received the first dose of Sinopharm vaccine (index date). The study period started on February 12, 2021, 14 days after receiving the first dose of the Sinopharm COVID-19 vaccine. Recipients of BNT162b2 (Pfizer-BioNTech), Ad26.COV2. (Johnson & Johnson-Janssen), the ChAdOx1 nCoV-19 (AZD1222; Oxford-AstraZeneca, and those for whom 1–13 days had elapsed since receipt of the first dose Sinopharm COVID-19 vaccine were excluded.

### Data sources

This is a linked health administrative database study. Three population-based data sources collecting Covid 19 related individual health information were used. Laboratory data (E-labs), SARS- CoV-2 severe disease/ hospitalization (Clinical data), and national vaccination register (vaccination data).

Laboratory data(E-labs) During the Covid 19 pandemic, the Ministry of health and social protection has introduced a laboratory information system (LIS) for the public and private sectors. E-labs is a national laboratory COVID-19 network for diagnostic specimens tested by Reverse Transcription Polymerase chain reaction (rt-PCR) in Morocco. LIS is critical for managing covid-19 tests, including the acquisition and dispatching of the medical material and kits and real-time epidemiological data generation, collection, processing, and sharing during the pandemic. The test result by rt-PCR for all individuals tested and included in this study was obtained. COVID-19 test result for individuals based on rapid antigen-based test kits or those clinically diagnosed is not registered in the E-labs registry.

Clinical data: COVID-19 associated severe/critical hospitalization are registered in a minimal clinical dataset made for public health surveillance. The clinical dataset is a national administrative dataset that includes SARS COV 2 severe/critical hospitalization data for a completed hospitalization episode.

### Vaccination data: National vaccination register

Vaccination histories were obtained from a common, unbiased electronic health record system. This dataset was based on a comprehensive and inclusive population-based list of target populations drawn up firstly for the entire population over the age of 17 based on a national identity card (NIC). This list is the basis of the national vaccination register (NVR). Vaccine receipt is associated with the registration of vaccine information in the NVR. The date when the COVID-19 vaccine generally became available, according to the age group of the recipients, was provided. Vaccine information included (vaccine date, vaccine type and vaccine dose).

National identity card number is used as the key to linking the three databases (NVR and E-labs registry and clinical dataset). This allowed us to generate a global database with National identity card as ID including all SARS-COV 2 rt-PCR tested patient during study period. vaccination status of all tested patients, negative rt-PCR tested patient, and positive rt-PCR tested patient with severe critical hospitalization was retained

### Study population

**The case group** were subjects with severe or critical SARS-CoV-2 infection confirmed by the rt-PCR positive test result based on laboratory. For each case, we considered the first rt-PCR -positive test for SARS-CoV-2 infection with associated COVID-19-related severe or critical hospitalization during the study.

### The control group

Controls are individuals who were tested negative for SARS-CoV-2 infection on an rt-PCR test; under the same conditions as the cases; based on laboratory data. For each control, we considered the first rt-PCR-negative test for SARS-CoV-2 infection during this period.

This strategy was used to control for potential bias due to repeat testing in rt-PCR-positive individuals seeking to check for infection clearance or bias arising from repeat testers among controls (persons with a higher level of healthcare-seeking behavior and presumably lower risk of infection).

We linked all rt-pcr SARS cov 2 tested patients (E-labs) to data for severe/critical SARS COV 2 hospitalized patient (Clinical data). We classified patients with positive rt-PCR test as being admitted to hospital with covid-19 if hospital admission dated between one and 14 days after the date of the SARS COV 2 rt-PCR sample.

- Severe and critical COVID-19 was defined according to WHO definition [3]. WHO defines severe COVID-19 as a SARS-CoV-2 infected individual with ‘oxygen saturation of <90% on room air and/or respiratory rate of >30 breaths per min in adults and/or signs of severe respiratory distress.

Critical COVID-19 is defined as a SARS-CoV-2-infected individual with ‘acute respiratory distress syndrome, sepsis, septic shock or other conditions that would normally require the provision of life sustaining therapies such as mechanical ventilation (invasive or non-invasive) or vasopressor therapy

### Exposure

Vaccination status was categorized based on the number of vaccine doses received and a number of days from vaccination to the SARS-CoV-2rT PCR.

- Unvaccinated
- Partial vaccination: included patients who received only the first dose of the vaccine and were tested 14 days and more after the first vaccination.
- Any time after 2^nd^ dose: Included individuals with receipt of a second dose of vaccine regardless of days before the index date.

Collected data included age and gender, rt-PCR test calendar date, rt-PCR test results, geographic location, and the 7-day moving average of the percentage of SARS-CoV-2 rt-PCR positive tests. The 7-day moving average of the percentage of SARS-CoV-2 rt-PCR positive tests was extracted from public health records; as a measure of SARS-CoV-2 circulation on the day of each rt-PCR test. Infection and vaccination statuses were both ascertained at the time of the PCR test.

### Ethical considerations

This real-world study was pre-registered (https://osf.io/at3yf) on Open Science Framework. The study was approved by the Rabat local ethic committee review board for biomedical research at Mohammed V University (N/21). A waiver of informed consent was granted for the study. Authorization N° A-RS-638/2021 from the National Commission for the Protection of Personal Data (CNDP) was obtained.

### Sample size

Although studies with large national datasets do not need to calculate the minimum sample size [4], we calculated the minimum sample size, considering that subgroup analysis will be needed to evaluate the vaccine protection effect (VE) for severe or critical infections. We estimated that VE is 80%, the population vaccination rate is approximate 50%. If the VE estimation accuracy is ±5%, and α = 0.05, according to the 1:1 matching of cases and controls, 2768 cases and 2768 controls are needed. Thus, 2768 in each of the two groups, with a total of 5536 subjects.

### Statistical analysis

Study samples were described using frequency distributions and measures of central tendency. We used a propensity score approach to control for observed confounding factors that might influence both group assignment and outcome [5]. Propensity score was defined as probability of receiving the vaccine given a potentially confounding covariates. Probability was estimated using a logistic regression model (using psestimate command in stata) with vaccine status as the dependent variable in relation to the following confounding observed covariates (sex, age, calendar days of the rt-PCR test; geographic location; and the 7-day moving average of the percentage of SARS-CoV-2–positive test) according to procedure described by Imbens and Rubin [6].

To adjust for differences between cases and controls, propensity score for vaccination is calculated for cases and controls. Then, we matched cases and controls in a one-to-one ratio by propensity score [7].

We used nearest-neighbor matching with a caliper of 0.2 SD. All non-matched patients are discarded. Imbalance after matching was checked using comparison between SARS-CoV-2–positive patients with negative controls. A standardized mean or proportion difference of more than 0.2 was considered to be noteworthy [8].

We used a test-negative design (TND) case–control study to evaluate the effectiveness of vaccination against confirmed SARS-CoV-2 infection. This design is distinct from a standard case–control [9] because the participants are selected before knowledge of the nature of their infection [10]. This is a widely accepted design for determining vaccine effectiveness in a population after the introduction of a vaccine [11,12].

We used univariate conditional logistic regression to calculate the odds ratio of testing positive among the vaccinated group versus the unvaccinated group [13]. Vaccine effectiveness and its associated 95% CI were then estimated using formula:

VE = (1 − Odds Ratio of vaccination among cases versus controls) x100%.

Our outcome of interest was vaccine effectiveness any time after receipt of the second dose and vaccine effectiveness by range time overtime after second dose receipt: (a) From 1st day to 30th days (during the first month after the second dose); (b) From 31th to 60th days (during the second month after the second dose); (C) From 61th to 90th days (during the third month after the second dose); (d) From 91st to120th days; (e) From 121th to 150th; and (f) beyond 150th day to 9th month.

We performed subgroup analyses by age group (⩽ 60 and > 60 years) and sex to determine vaccine effectiveness in various population subgroups. Effectiveness was calculated for each subgroup any time after the second dose, and in three range time [14].

The same analysis was performed in sensitivity analyses. We used unconditional logistic regression to assess the odds ratio of testing positive among the vaccinated group versus the unvaccinated group after adjustment for sex, age, calendar days of the rt-PCR test; geographic location; and the 7-day moving average of the percentage of SARS-CoV-2–positive test.

*P* values were calculated to test the significance of the difference within each subgroup. A *P* value less than 0.05 was considered significant. For all point estimates of vaccine effectiveness, we calculated 95% CIs.

## Results

Among 12884 persons who tested positive and 12885 propensity score-matched control participants, the median age was 62 years. We identified 25,768 matched pairs of patients who were tested for SARS-CoV-2 infection between 1 February 2021 and 01 October 2021 (1:1 ratio of positive and negative test results for SARS-CoV-2 infection). The median age was 62 years among those who tested negative and 63 years among those who tested positive for SARS-CoV-2 infection; in both groups, 47.2% of participants were female. Among those who tested positive, 1015 (7.9%) had been vaccinated, and among those who tested negative, 2935 (22.8%) had been vaccinated. Baseline characteristics of the study population are shown in Table 1.

**Table 1:** Baseline characteristics of individuals tested positive for SARS-COV-2 infection by rt-PCR test and Propensity Score–Matched control participants who tested negative for SARS-COV-2 infection.

Characteristics	Overall (n = 25768)	Tested Negative (n = 12884)	Tested Positive (n = 12884)	Standardized mean difference
Median (IQR) age at specimen collection date, years	62 (49–73)	62 (49–73)	63 (50–72)	0.002
**Age at specimen collection date, years:** NA				
	3645 (14.2)	1749 (13.6)	1896 (14.7)	
Age 41–60 years, n (%)	8190 (31.8)	4296 (33.3)	3894 (30.2)	
Age ≥ 60 years, n (%)	13933 (54.1)	6839 (55.1)	7094 (55.1)	
**Gender:**				0.017
Female, n (%)	12,169 (47.2)	6140 (50.5)	6029 (49.5)	
Male, n (%)	13599 (52.8)	6744 (49.6)	6855 (50.4)	
**Month of specimen collection:**				0.004
February 2021	183 (0.7)	130 (1.0)	53 (0.4)	
March 2021	512 (2.0)	357 (2.8)	155 (1.2)	
April 2021	996 (3.9)	324 (2.5)	672 (5.2)	
May 2021	806 (3.1)	294 (2.3)	512 (4.0)	
June 2021	1263 (4.9)	686 (5.3)	577 (4.5)	
July 2021	3643 (14.1)	1923 (14.9)	1720 (13.3)	
August 2021	11999 (46.6)	5976 (46.4)	6023 (46.7)	
September 2021	5686 (22.1)	2905 (22.5)	2781 (21.6)	
**October 2021**	680 (2.6)	289 (2.2)	391 (3.0)	
**The 7-day moving average percentage SARS-CoV-2 positive test (Median (IQR))**	18.12 (10.02–21.71)	18.12 (10.02–21.71)	18.12 (9.81–21.71)	0.003
**Vaccine Status**				NA
Unvaccinated (%)	21818 (84.7)	9949 (77.2)	11869 (92.1)	
Partially vaccinated (%)	448 (1.7)	285 (2.2)	163 (1.3)	
Any time after second dose (%)	3502 (13.6)	2650 (20.6)	852 (6.6)	

IQR = interquartile range. NA: Not appliquable.

**Fig 1:**
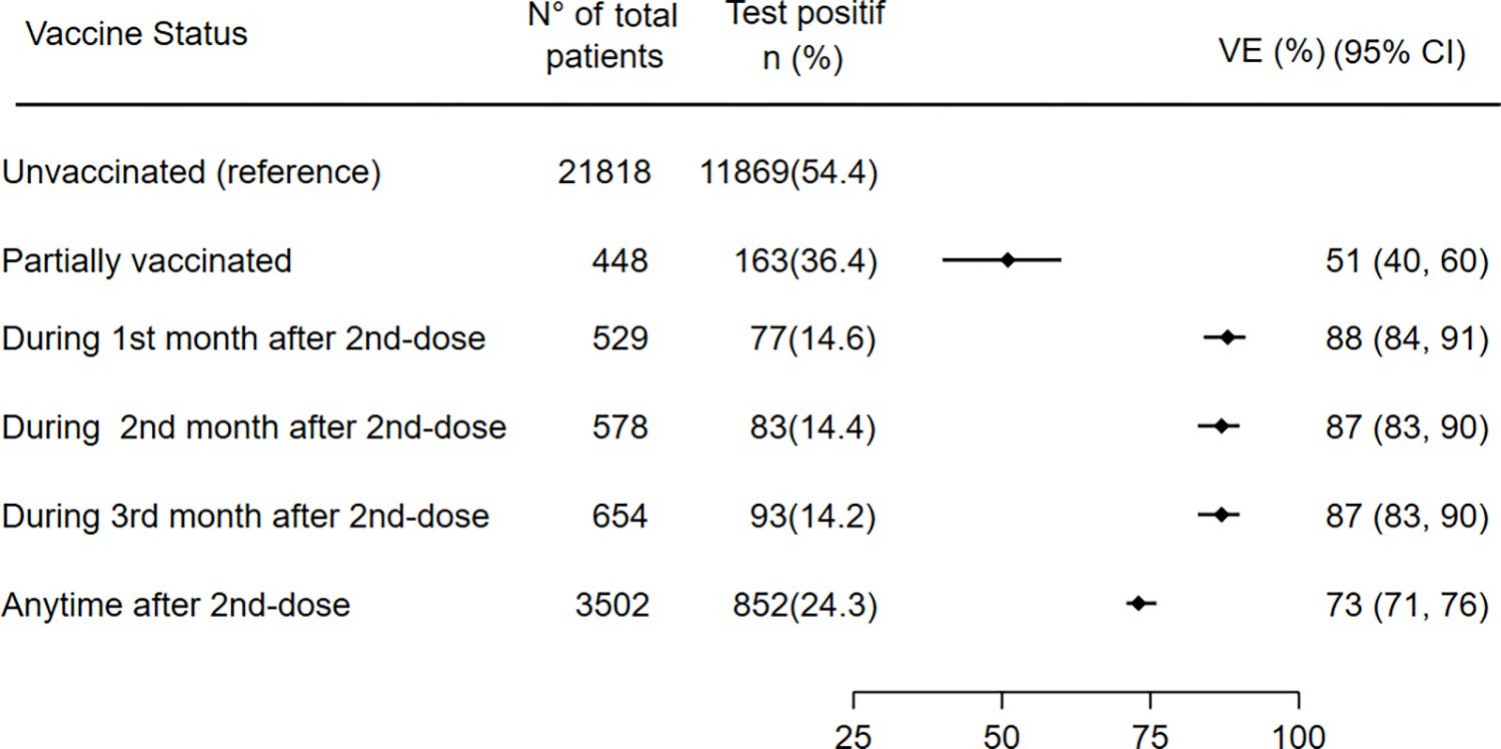
Estimates Vaccine effectiveness of inactivated vaccine BBIBP-CorV against COVID-19-associated severe or critical hospitalization according to vaccination status and by time since vaccination.

**Fig 2:**
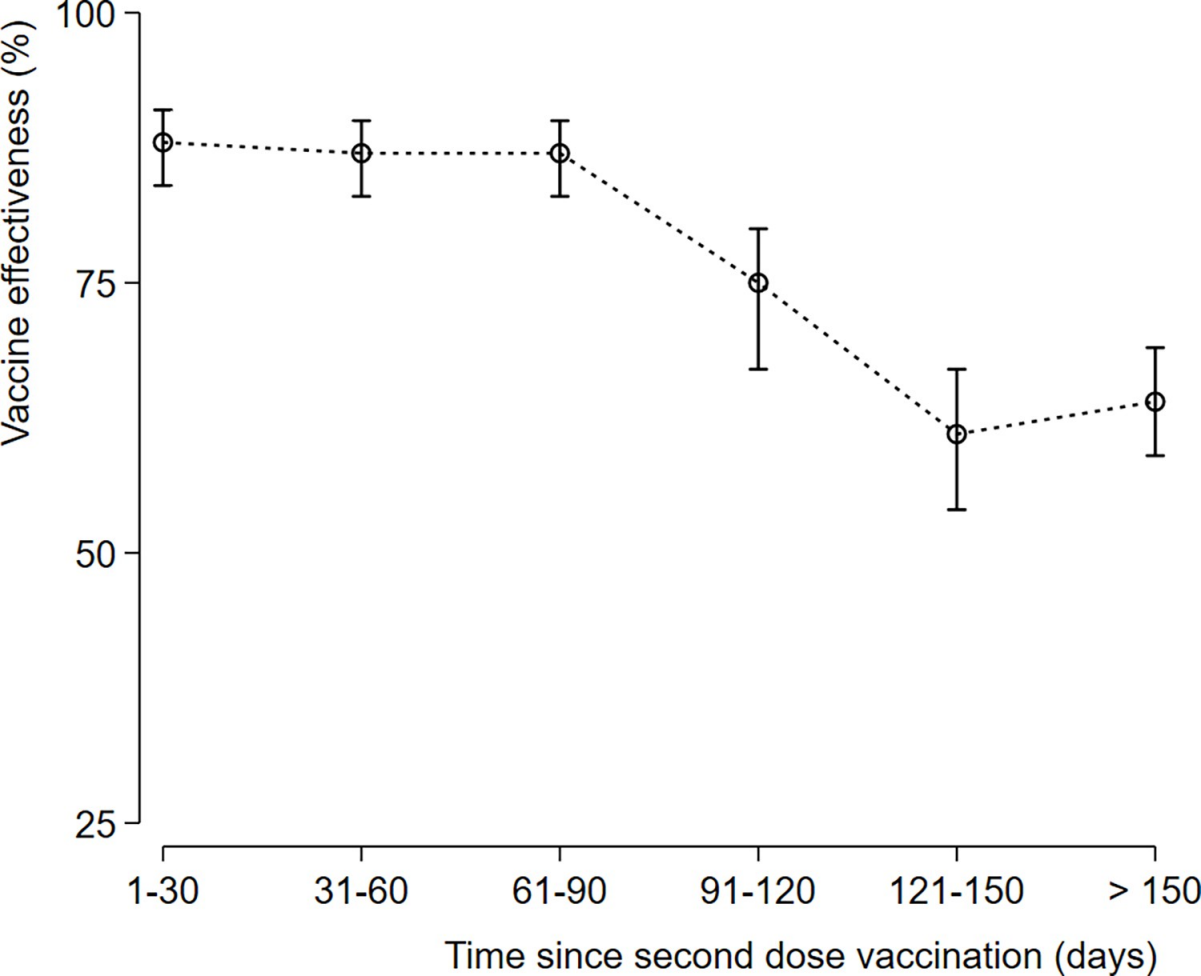
Vaccine Effectiveness of inactivated vaccine BBIBP-CorV against COVID-19-associated severe or critical hospitalization over time since second dose vaccination. Vaccine Effectiveness at each Time Point: At (1–30) time point; VE 88% (95% CI: 84–91), At (31–60) time point; 87% (95% CI: 83–90), At (61–90) time point; 87% (95% CI: 83–90), At (91–120) time point; 75% (95% CI: 67–80), At (121–150) time point; 61% (95% CI: 54–67), Beyond 150 days’ time point; 64% (95% CI: 59–69).

As a function of time after vaccination of second dose vaccination, vaccine effectiveness among persons who had received the second dose 1–30 days earlier was 88% (95% CI, 84–91), 87% (95% CI: 83–90) among those who had received it 31–90 days earlier, 75% (95% CI: 67–80) among those who had received it 91–120 days earlier, 61% (95% CI: 54–67) among those who had received it 121–150 days earlier, 64% (95% CI: 59–69) among those who had received it ≥150 days earlier. The overall vaccine effectiveness any time after the second dose was 73% (95% CI: 71–76). Estimates Vaccine effectiveness against COVID-19 associated severe or critical hospitalization according to vaccine status and by time since vaccination is shown in Figs 1 and 2.

**Fig 3:**
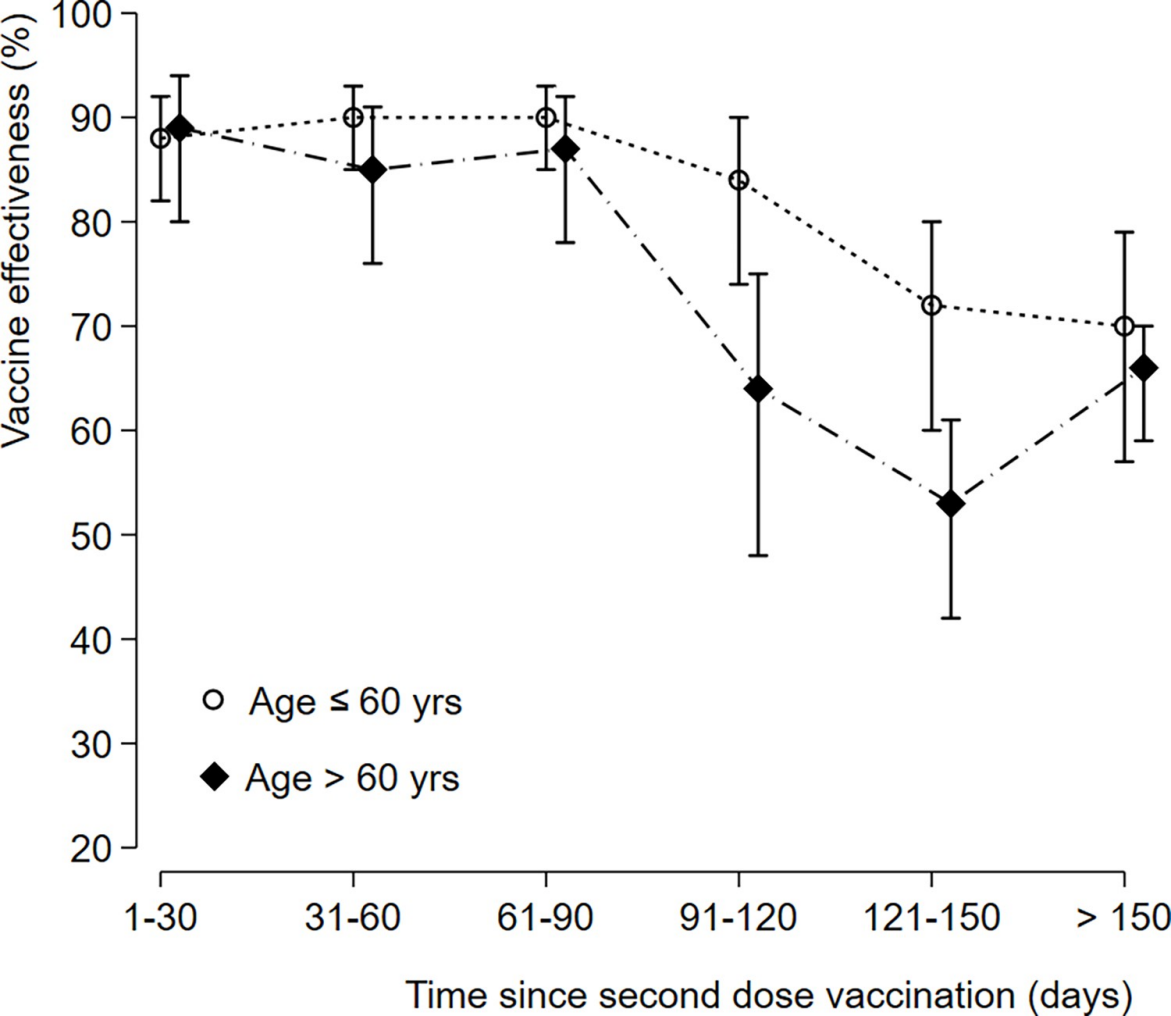
Vaccine effectiveness of inactivated vaccine BBIBP-CorV against COVID-19-associated severe or critical hospitalization over time since second dose vaccination according to age subgroup. Vaccine Effectiveness at each Time Point in age subgroup ≥ 60 years old: VE at (1–30) time point; 80% (95% CI: 80–94).VE at (31–60) time point; 85% (95% CI: 76–91). VE at (61–90) time point; 87% (95% CI: 78–92). VE at (91–120) time point; 64% (95% CI: 48–75). VE at (121–150) time point; 53% (95% CI: 42–61). VE Beyond 150 days’ time point; 66% (95% CI: 59–70). Vaccine Effectiveness at each time point in age subgroup < 60 years old: VE at (1–30) time point; 88% (95% CI: 82–92). VE at (31–60) time point; 90% (95% CI: 85–93). VE at (61–90) time point; 90% (95% CI: 85–93). VE at (91–120) time point; 84% (95% CI: 74–90). VE at (121–150) time point; 72% (95% CI: 60–80). VE Beyond 150 days’ time point; 70% (95% CI: 57–79).

**Fig 4:**
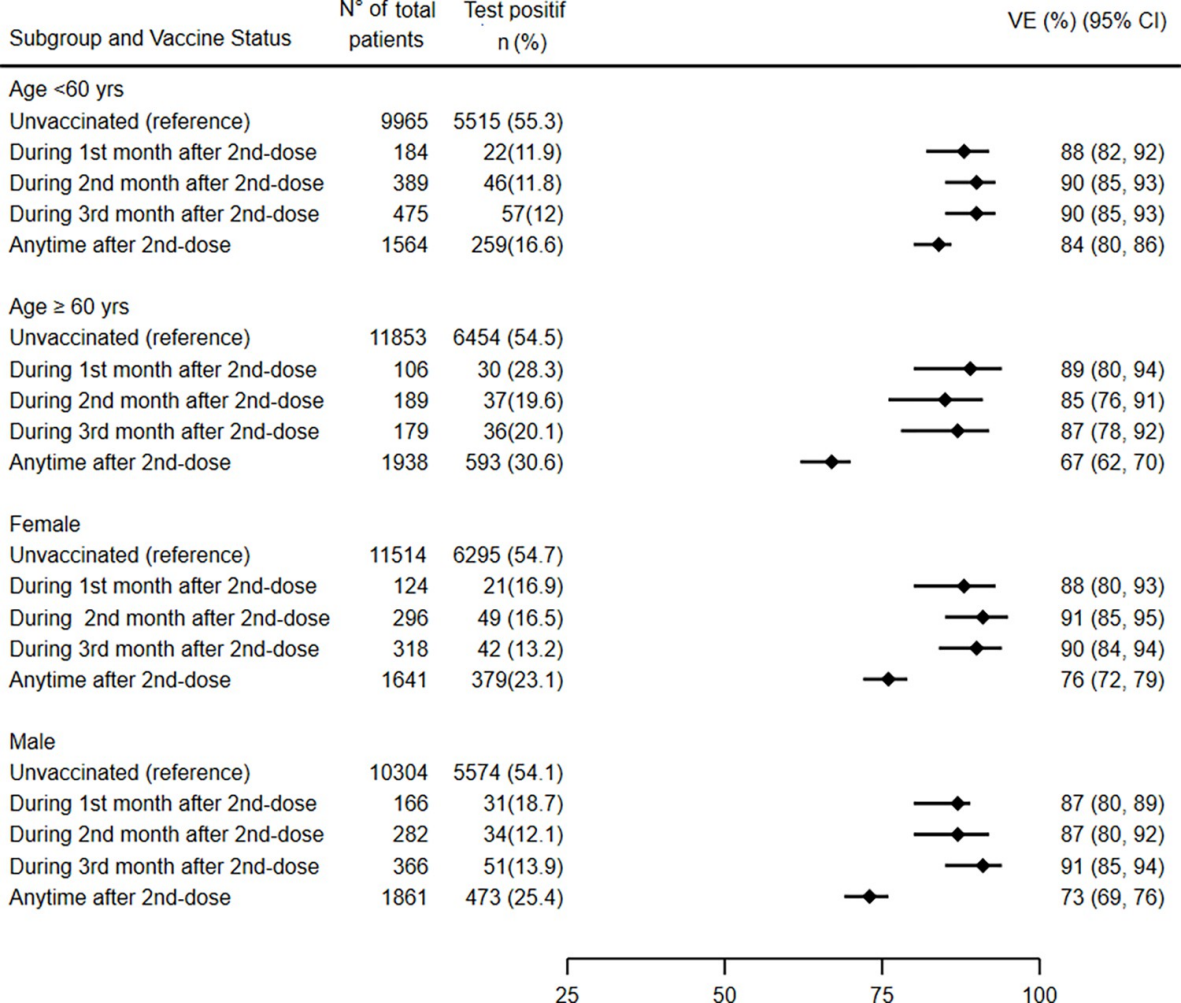
Estimates Vaccine effectiveness of inactivated vaccine BBIBP-CorV against COVID-19-associated severe or critical hospitalization according to age and gender over time since second dose vaccination.

Concerning vaccine effectiveness of inactivated vaccine BBIBP-CorV against COVID-19 associated severe or critical hospitalization over time since vaccination of the second dose in age subgroup. a) Concerning age subgroup ≥ 60 years old: VE at (1–30) time point was 80% (95% CI: 80–94), at (31–60) time point was 85% (95% CI: 76–91), at (61–90) time point was 87% (95% CI: 78–92), at (91–120) time point was 64% (95% CI: 48–75, at (121–150) time point was 53% (95% CI: 42–61), and beyond 150 days’ time point was 66% (95% CI: 59 to 70). b) Concerning age subgroup < 60 yrs old: VE at (1–30) time point was 88% (95% CI: 82 to 92), at (31–60) time point was 90%(95% CI: 85–93), at (61–90) time point was 90% (95% CI: 85–93), at (91–120) time point was 84% (95% CI: 74–90), at (121–150) time point was 72% (95% CI, 60–80), and beyond 150 days’ time point was 70% (95% CI: 57–79). Any time after the second dose, vaccine effectiveness against severe critical hospitalization in persons younger than 60 years compared with those aged 60 years and older was: 84% (95% CI: 80–86) and 67% (95% CI: 62–70) respectively. (Figs 3 and 4). In the gender subgroup effectiveness was similar between men and women (Fig 4). Estimates of vaccine effectiveness after the second dose as a function of time since second dose vaccination according to subgroup are shown in Figs 3 and 4.

Sensitivity analyses using a simple logistic regression were consistent with the primary analysis based on propensity matching. Results of sensitivity analyses are presented in Tables 1 and 2 in the S1 Annexe.

## Discussion

We provide estimates of the vaccine effectiveness (VE) of the administration of the Sinopharm vaccine in a countrywide mass vaccination campaign for the prevention of severe and critical COVID-19 associated hospitalization in the Kingdom of Morocco. Among fully immunized persons, the vaccine effectiveness at any time during the first 9 months after the second dose was 73%. Due to the longer follow-up time for this study since vaccine rollout, this study provides information on the stability profile of Sinopharm vaccine effectiveness. Estimates of COVID-19 inactivated Sinopharm vaccine effectiveness (VE) decline on the time elapsed since the second dose.

Our results showed that the effectiveness of Sinopharm vaccine against severe COVID-19 associated hospitalizations reached a maximum level during the first month, and remains high and durable during the three months after the second dose (87–90%), declines little in the fourth month (75%), and stabilizes around 60% beyond the fifth month. In a short time since vaccine rollout, our results are not consistent with the results of phase III clinical trial given the small number of incident severe cases [15], but consistent with previous studies [16]. The nine months’ follow-up revealed that the effectiveness of the vaccine started to decline after three months, and that the effectiveness against critical care admissions dropped below 60% after six months. Similar results were reported from another study that showed that the effectiveness against severe outcomes of COVID-19 was 80%, 92% and 97% against hospitalization, critical care admission, and death respectively and that long-term effectiveness of BBIBP-CorV vaccine decline below 75% after six months [17]. Vaccine effectiveness results differ in age subgroup analysis, with those aged 60 years or older showing an earlier and more evident decline beyond the third-month post-vaccination. Due to immune-senescence and comorbidities, older people are more susceptible to infections and respond less well to vaccination [18].

Other studies also demonstrated a gradual waning of the immune response and vaccine effectiveness within months after vaccination with the BNT162b2 vaccine. This waning effectiveness of vaccines over time is likely explained by the viral evasion of vaccine induced immune response through antigenic changes in newer variants of the SARS-CoV-2 virus, as well as the decline in antibody levels that are evident from studies which have shown a reduction in anti-S antibodies three weeks after the second dose of vaccination [19]. Shrotri et al showed a waning of S-antibody levels in infection-naive individuals over a 3–10-week period after a second dose of 2 others anti Sars cov 2 vaccine consistent with the decline in Spike-antibody and neutralizing antibody levels observed after infection, although memory B-cell populations appear to be maintained [20]. the clinical implications of waning antibody levels post-vaccination lead to an increase in serious covid 19 disease. Then; it remains crucial to establish S-antibody thresholds associated with protection against clinical outcomes.

With continued global incidence, and potential for more transmissible SARS-CoV-2 variants, data on longer-term vaccine efficacy and antibody dynamics are essential for clarifying the need for further booster doses.

Due to the nature of real-world effectiveness studies, they can be subject to selection bias, confounding factors, and missing data, therefore requiring careful study design. A test-negative design case-control study was in the general population, which may be subject to collider bias. Due to the observational study design, selection bias and confounding effects were inevitable limitations. Then, this analytical approach was implemented to reduce potential bias due to variation in the epidemic phase, gradual vaccination and other confounders [21]. The test-negative design has been used extensively to estimate VE among medically attended influenza virus illness and is believed to minimize biases associated with access to vaccines and healthcare-seeking behaviors, and recommended by the WHO interim guidance [22,23].

Limitations of our study include looking only at, critical care hospitalization as outcomes. We did not estimate the effectiveness of the vaccine against other outcomes, such as symptomatic infections; hospitalization, organ injury or death. we primarily investigated the severe and hospitalized COVID-19 cases, which are a better indicator of vaccine effectiveness for public health relevance. Second, we adjusted for factors, such as age, sex, calendar days of the rt-PCR test; geographic location; and the 7-day moving average of the percentage of SARS-CoV-2–positive test., but we did not account for other factors that may have influenced the outcomes such as obesity, comorbidities, smoking, occupation, and clinical data. The propensity score for each study participant is based on the available measured patient characteristics, and unadjusted confounding may still exist if unmeasured factors influenced vaccine status. Therefore, using fewer variables in the propensity score model reduces the likelihood of effectively adjusting for confounding. Another limitation of our study is though the decline in vaccine effectiveness over time were analyzed we could not rule out the effect of the changes in the circulating variants on the waning of vaccine effectiveness over months due to lack of linkage to sequencing.

In conclusion, in accordance with the results of phase III clinical trials, the licensed Sinopharm vaccine is highly protective against SARS-CoV-2 in real conditions. In addition, this study confirms the durability of the protection. VE depends not only on the effectiveness of the vaccine itself but also on factors such as the age of the recipient and time since the latest dose.

This study of vaccine effectiveness focuses on the specific subset of cases—severe or critical—with the greatest burden on health systems, regardless of their level of maturity, and requires the most attention for vaccine effectiveness evaluations. The elderly population is more vulnerable and at greater risk of immune depletion overtime after full immunization and deserves special attention. Therefore, maintaining the stability of their protection, through either continued social distancing or active vaccination, must be a priority. Governments may face declining public confidence in the vaccines used in their country, post-licensure observational evaluations are important to inform policy decisions about vaccine introduction, to optimize vaccine implementation programs and to provide evidence in use support of vaccines and investments by governments and donors, and above all to convey a convincing message to the population. By combining these features, the present study has generated new evidence that helps in making informed decisions.

## Supporting information

S1 Annexe S1 and S2 Tables. (DOCX)

S2 Annexe Additional data file. (XLSX)
