# Correction: Long term effectiveness of inactivated vaccine BBIBP-CorV (Vero Cells) against COVID-19 associated severe and critical hospitalization in Morocco

**DOI:** 10.1371/journal.pone.0316865

**Published:** 2024-12-30

**Authors:** Jihane Belayachi, Majdouline Obtel, Abdelkader Mhayi, Rachid Razine, Redouane Abouqal

The following information is missing from the Acknowledgments section: The authors would like to thank the Académie Hassan II des Sciences et Techniques for its valuable contribution in covering the publication fees.

There are errors in the author affiliations. The correct affiliations are as follows:

Jihane Belayachi^1,2^, Majdouline Obtel^2,3^, Abdelkader Mhayi^4^, Rachid Razine^2,3^, Redouane Abouqal^1,2^

**1** Acute Medical Unit, Ibn Sina University Hospital, Rabat, Morocco, **2** Laboratory of Biostatistics, Clinical, and Epidemiological Research, Faculty of Medicine and Pharmacy, Department of Public Health, Mohammed V University in Rabat, Rabat, Morocco, **3** Laboratory of Community Health (Public Health, Preventive Medicine and Hygiene), Faculty of Medicine and Pharmacy, Department of Public Health, Mohammed V University in Rabat, Rabat, Morocco, **4** Department of Informatics, Ministry of Health and Social Protection, Rabat, Morocco.
