# Supplementary material for: Long term effectiveness of inactivated vaccine BBIBP-CorV (Vero Cells) against COVID-19 associated severe and critical hospitalization in Morocco
Source: PLoS One. 2022 Dec 7;17(12):e0278546. doi: 10.1371/journal.pone.0278546 (PMC9728886; doi:10.1371/journal.pone.0278546)
Supplement: S1 Annexe — (DOCX) [file pone.0278546.s001.docx]

**s-Table 1:** Estimates of overall and according to age and gender Vaccine effectiveness of inactivated vaccine BBIBP-CorV against COVID-19-associated severe or critical hospitalization over time since second dose vaccination: Sensitivity analysis using simple logistic regression

| **Vaccine status** | **VE (95% CI)** |
| --- | --- |
| **Overall vaccine status** |  |
| Unvaccined |  |
| Partially vaccined | 53 (43 ; 61) |
| During 1^st^month after 2-nd dose | 86 (82 ; 89) |
| During 2 month after 2-nd dose | 87 (82 ; 89) |
| During 3 month after 2-nd dose | 87 (82 ; 89) |
| Any time after second dose | 74 (72 ; 76) |
| **Subgroup and vaccine status** |  |
| **Age<60 years** |  |
| Unvaccined |  |
| During 1^st^month after 2-nd dose | 88 (83 ; 91) |
| During 2 month after 2-nd dose | 89 (85 ; 92) |
| During 3 month after 2-nd dose | 89 (86 ; 92) |
| Any time after second dose | 84 (82 ; 86) |
| **Age≥60 years** |  |
| Unvaccined |  |
| During 1^st^month after 2-nd dose | 82 (73 ; 88) |
| During 2 month after 2-nd dose | 80 (71 ; 86) |
| During 3 month after 2-nd dose | 80 (71 ; 86) |
| Any time after second dose | 64 (60 ; 68) |
| **Female** |  |
| Unvaccined |  |
| During 1^st^month after 2-nd dose | 86 (80 ; 91) |
| During 2 month after 2-nd dose | 88 (83 ; 92) |
| During 3 month after 2-nd dose | 88 (83 ; 91) |
| Any time after second dose | 75 (72 ; 78) |
| **Male** |  |
| Unvaccined |  |
| During 1^st^month after 2-nd dose | 85 (80 ; 89) |
| During 2 month after 2-nd dose | 84 (78 ; 88) |
| During 3 month after 2-nd dose | 86 (81 ; 89) |
| Any time after second dose | 73 (69 ; 76) |

VE (95% CI): vaccine effectiveness (95% confidence interval). Sensitivity analysis with simple logistic regression was used to assess the odds ratio of testing positive among the vaccinated group versus the unvaccinated group after adjustment for sex, age, calendar days of the rt-PCR test; geographic location; and the 7-day moving average of the percentage of SARS-CoV-2–positive test.

**s-Table 2:** overall, and age subgroup Vaccine effectiveness of inactivated vaccine BBIBP-CorV against COVID-19-associated severe or critical hospitalisation over time since second dose vaccination: Sensitivity analysis using simple logistic regression

| Time since second vaccination | **Vaccine Effectiveness (95% CI)** | | |
| --- | --- | --- | --- |
|  | Overall vaccine status | Age < 60 years | Age ≥ 60 years |
| From 1 to 30 Days | 86 (82 ; 89) | 88 (83 ; 91) | 82 (73 ; 88) |
| From 31 to 60 Days | 86 (82 ; 89) | 89 (85 ; 92) | 80 (71 ; 86) |
| From 61 to 90 Days | 87 (82 ; 89) | 89 (86 ; 92) | 80 (71 ; 86) |
| From 91 to 120 Days | 73 (66 ; 79) | 83 (75 ; 88) | 62 (48 ; 72) |
| From 121 to 150 Days | 64 (58 ; 69) | 75 (66 ; 82) | 58 (49 ; 65) |
| More than 151 Days | 66 (61 ; 70) | 71 (61 ; 78) | 64 (58 ; 69) |

**(95**% confidence interval**)**. Sensitivity analysis with simple logistic regression was used to assess the odds ratio of testing positive among the vaccinated group versus the unvaccinated group after adjustment for sex, age, calendar days of the rt-PCR test; geographic location; and the 7-day moving average of the percentage of SARS-CoV-2–positive test.
